# Profile-based short linear protein motif discovery

**DOI:** 10.1186/1471-2105-13-104

**Published:** 2012-05-18

**Authors:** Niall J Haslam, Denis C Shields

**Affiliations:** 1 Complex and Adaptive Systems Laboratory, University College Dublin, Dublin, Ireland; 2 Conway Institute of Biomolecular and Biomedical Research, University College Dublin, Dublin, Ireland; 3 School of Medicine and Medical Sciences, University College Dublin, Dublin, Ireland

**Keywords:** Protein-protein interactions, Motif discovery, Peptide binding, Short linear motifs, Mini-motifs, SLiMs

## Abstract

**Background:**

Short linear protein motifs are attracting increasing attention as functionally independent sites, typically 3–10 amino acids in length that are enriched in disordered regions of proteins. Multiple methods have recently been proposed to discover over-represented motifs within a set of proteins based on simple regular expressions. Here, we extend these approaches to profile-based methods, which provide a richer motif representation.

**Results:**

The profile motif discovery method MEME performed relatively poorly for motifs in disordered regions of proteins. However, when we applied evolutionary weighting to account for redundancy amongst homologous proteins, and masked out poorly conserved regions of disordered proteins, the performance of MEME is equivalent to that of regular expression methods. However, the two approaches returned different subsets within both a benchmark dataset, and a more realistic discovery dataset.

**Conclusions:**

Profile-based motif discovery methods complement regular expression based methods. Whilst profile-based methods are computationally more intensive, they are likely to discover motifs currently overlooked by regular expression methods.

## Background

In protein-protein interaction networks, hub proteins are defined as those that interact with a number of other proteins, either simultaneously or at different times. Whilst domain-domain interactions are important for stable interactions, rapid low affinity interactions mediated by short linear motifs are important for more transient interactions, for example in signal transduction [1,2]. Short linear motifs (SLiMs) are typically 3–10 residue stretches of a protein sequence, with two or more non-wildcard positions that independently mediate a range of functions. They may be involved in ligand binding, modification, targeting and cleavage [3], all of which are important in driving cell signaling [1,4]. Motifs can act in a coordinated and co-operative manner to exhibit functional regulatory complexity within the cell [5]. Therefore, the known repertoire of protein modules needs to be expanded to include smaller functional sites like SLiMs, in addition to well-characterised domain modules. This will advance understanding of the fundamental mechanisms that drive protein-protein interactions.

The current (2012) release of the Eukaryotic Linear Motif (ELM) database lists 174 experimentally validated short protein motifs [3], and the MiniMotif Database contains around 5,000 predicted motifs [6]. Databases dealing with motifs containing sites for post-translational modificatoins (PTMs) alone list in the region of tens of thousands of motifs [7,8]. Surveys have shown that up to 30% of the human proteome is disordered [9]. Disordered regions are known to be rich in linear motifs [10,11]. Given the relatively low number of motifs so far identified, it is clear that much work is still to be done [10]. Therefore, it is imperative that new tools are developed to meet this challenge.

One approach to discovering short functional sequence motifs is to apply computational tools to find a motif that is over-represented among a group of evolutionarily unrelated sequences that have a related function (e.g. they bind a common interacting protein). In DNA motif discovery, profile-based methods have been very successful in the identification and classification of transcription factor and promoter binding sites [12]. However, profile-based methods have not been as widely used in the search for protein motifs since the first publication of the MEME tool for motif discovery [13]: computational methods for the discovery of protein motifs [14,15] have focused on regular expression over-representation, whilst profile-based discovery programs such as MEME [16] have been largely confined to DNA analysis.

Profile-based methods aim to describe the motif in terms of the relative frequencies of amino acids at each position. The regular expression [DE] allows Aspartic and Glutamic acid at that position, and does not define the relative frequency with which they are found at that position. However, in a profile-based definition it is possible to state that Aspartic acid is present 70% of the time, and Glutamic acid 30%. This allows a more refined definition of the motif. Various methods have been proposed to define a profile of a linear motif – summarized in [17]. Regular expressions are commonly used to attempt to capture the relevant sequence information about a linear motif [14]. Such representations of motifs have been favoured by biologists as they are sometimes more intuitive than profiles. However, profile-based representations may present certain potential advantages: they can sometimes provide a richer and more accurate representation of certain motifs, and can have a visual representation (using a “sequence logo” [18]) that is often more easily understood than some of the more complex regular expressions for highly redundant motifs.

The shortness of SLiMs makes their discovery difficult, because of the resulting difficulty in distinguishing true positive from false positive matches. This difficulty is further compounded by the degree of variation between instances of a linear motif. Thus, careful evaluation in a realistic setting of biological discovery is needed to determine if methods are useful in practice. Many motifs lie in disordered regions of proteins, and the motifs are often distinguished by greater evolutionary conservation among orthologues; this property allows a focus on evolutionarily conserved residues to increase the chance of discovering novel motifs [19]. Focussing on regions conserved in orthologues by masking out non-conserved disordered residues, and discounting motifs recurrent simply amongst homologous rather than unrelated proteins, have been shown to greatly improve the performance of regular expression based methods to both identify true instances of known motifs, and to discover novel motifs [14,15]. In this paper, we investigate to what extent the application of evolutionary weighting and masking of protein sequences can improve the performance of profile-based methods to discover short linear protein motifs.

## Methods

A set of protein sequences is used as the query set. These sequences, or a subset of them, are believed to contain a common motif responsible for a functional activity. The motif is likely to be relatively conserved between orthologues of the proteins in other related species, in contrast to generally unconserved surrounding disordered regions of the protein. We used the relative local conservation scoring system described in Davey et al. 2009 [19] to mask out unconserved residues of the query sequences before submitting the sequences to the MEME program to discover over-represented SLiM profiles amongst the sequences [16]. In addition, we used the SLiMBuild algorithm from SLiMFinder to produce weightings of the relatedness of the query sequences to each other [15].

Additional masking of the query sequences to remove transmembrane regions and domains, taken from UniProt annotation [20], was performed in order to increase the likelihood of identifying linear motifs in the query sequences, by eliminating such sources of high-scoring false positives.

Previous work by Fuxreiter et al. has shown that disordered regions are enriched for short linear motifs [21]. This has been confirmed by a separate analysis of experimentally validated motifs from the ELM database [22]. Both indicate that the residues that comprise the motif are likely to have high disorder propensities as compared to the flanking regions. A cutoff of 0.3 ensures a balance between reducing the search space excessively whilst removing regions of the protein known to be ordered. From Davey et al. [22] 82% of known motifs have a disorder score over 0.3 the cut-off used in this analysis.

### Orthology detection and alignment generation

In order to generate alignments for the proteins in the benchmark dataset, we used the series of metazoan Ensembl whole genomes downloaded in March 2010 [23]. We follow the method used in the Gopher orthologous protein identification and alignment algorithm described in Edwards et al. [24]. Each query sequence in the set was searched using BLAST (masking out low complexity regions) against the metazoan proteome at an expectation threshold of e = 10-4. The set of hits from this search was then used to search against the database again at a relaxed threshold of e = 10, but without complexity filtering. Sequences at this stage had to have 40% global similarity to the original query for inclusion. The most similar sequence for each species was retained for inclusion in the alignment. Multiple sequence alignments were then generated using the MUSCLE program [25].

We adopted the treatment of evolutionary information previously developed and evaluated for SLiM discovery [15,26], since the problem of treating evolutionary information is likely to be very similar for both profile and regular expression discovery of linear motifs. Improving public orthology resources such as those of Ensembl [23] may prove useful in future implementations of the method, accelerating calculations.

### Relative local conservation

Disordered regions have different patterns of conservation compared to structured regions of protein sequences. Therefore, traditional multiple sequence alignments are not particularly informative when analyzing the conservation of proteins that include disordered regions, since the pattern of conservation is dominated by the pattern of order and disorder across the sequence. To overcome this, we applied relative local conservation (RLC) masking [19], which assesses the conservation of disordered residues relative to adjacent disordered residues, which we summarise briefly below.

Residues are marked as belonging to one of two structural states: ordered or disordered; using the IUPred disorder prediction program (short setting, threshold 0.3) [27]. Only residues within, 25 residues to either side that were in the same structural state were compared. Then the residues in each column of the alignment were scored for conservation.

The RLC is calculated for each residue using a multiple sequence alignment of the protein with its orthologues. We used Ensembl release 60 metazoan proteomes to generate the alignments [23]. Then the residues in each column of the alignment were scored for conservation. For each residue, this was compared against the background mean conservation in a window of 25 residues to either side of the residue across the sequence. Strongly conserved residues are given a high score with more variable residues given a low score [19]. The RLC score for the residue is calculated (in the manner of a standard Z-score) by subtracting the background mean conservation and normalizing by dividing by the standard deviation of the window. This results in normalized scores that are comparable between residues in different protein sequences, irrespective of differences in divergence patterns. Scores above 0 indicate above average relative local conservation.

There are a number of alternative search strategies possible for exploiting information on conservation. One method would be to use absolute conservation level levels relative to orthologues from defined species. However, previous work has shown that for discovery of motifs in predominantly disordered intracellular eukaryotic protein regions, the relative local conservation compared to nearby residues of the motif is more powerful [19]. Accordingly, we adopted this approach, since the dataset to which we were applying this analysis are primarily intracellular motifs. Extracellular motifs, where there is much less protein disorder, may well benefit from other approaches.

### Evolutionary weighting

The aim of evolutionary weighting is to appropriately reduce the statistical support for motifs that are over-represented because of large-scale sequence homology (identity by descent) among a subset of the proteins investigated, rather than because of convergent evolution to a common motif among unrelated proteins. This is achieved by grouping query sequences into unrelated protein clusters (UPCs). Proteins within a given search set were analysed iteratively by BLAST (E-value threshold of 10–3) to determine relatedness. Proteins determined by BLAST to be related were grouped into a UPC. Each protein in the cluster is not obviously related to any protein in another cluster. While a similar correction could be more simply achieved by only choosing one of the related proteins in the motif search [14] the approach taken here is favoured because short motifs typically evolve faster than domains [28], so that often only a subset of the related proteins may possess the motif. The weighting of sequences occurs after the assignment of proteins into UPCs. This is to ensure that the similarity is calculated based on the full sequence and not the masked regions which may be misleading in homology assignment.

### Profile-based discovery of motifs using meme

MEME uses expectation maximization methods to identify over-represented motifs in the query set [16]. The program was presented with the unmasked dataset, the masked dataset, the weighted dataset and the masked and weighted dataset to judge the impact of each of these methods on the performance. The evolutionary weighting was calculated using SLiMBuild [24] and the masking by using the RLC masking from SLiMFinder [15] as described above.

The datasets were given as the input to MEME running with the expectation that there would be zero or more motif instances in the query set. The minimum length of the motifs was set at 3 and the maximum length at 10. Low complexity filters were switched off. The profile search is carried out after the sequences are filtered for disorder; there is no lower limit on the length of disordered sequence considered, except that the motif discovery methods require motifs of at least three residues in our analysis.

MEME has an option to weight sequences. To apply evolutionary weighting, we incorporated knowledge regarding the distances among sequences generated in the UPC building process to weight the sequences appropriately. A minimum spanning tree normalization was used to weight sequences as described in SLiMDisc [26]. This is derived from a matrix of the sequence similarity of the sequences. The distances ranged between zero and 1, where zero indicates no similarity, and 1 indicates identical sequences. The weights for each sequence (W) were then estimated from the average distances to all other sequences (S_d_) and the number of other sequences (N), as follows:

$$\;W=\left(1-\bar{S_{d}}\right)/N$$

Consider the sequences A, B, C, and D. If A and B are 50% similar to each other and 0% similar to C and 25% similar to D, and D and C are 0% similar to each other. A and B have a distance 0.5; A and C have a distance of 1; and A and D have a distance of 0.75. A will have a score of 0.56, B will have a score of 0.56, C will have a score of 1 and D will have a score of 0.69. This ranks the sequences in order of their similarity to all other sequences.

### Datasets

For the purposes of benchmarking the effectiveness of the evolutionary weighting and relative local conservation masking method we took datasets from a number of papers in the field, to facilitate comparisons among methods. The first dataset was from [14]. It used a gold standard literature based dataset from the ELM database [29].

The second dataset is a more realistic test of the normal operation of the program using protein-protein interaction data downloaded from the Human Protein Reference Database (HPRD) [30] taken from [19]. The aim of using this dataset was to test the ability of the program to uncover motifs in a dataset that is known to be noisy. Both datasets are available from the authors of the original manuscripts and are included in the supplementary material.

## Results

### Evolutionary weighting and masking out non-conserved regions improves discovery of motifs in disordered regions in a standard dataset

Table 1 shows the results for the ELM dataset of known motifs, occurring mainly in disordered regions of proteins. This compares MEME’s ability to recover known motifs under different conditions with a regular expression method, SLiMFinder. Regular expression approximations of the profiles are shown in Table 1 to facilitate comparison of the results. Overall, it is clear that the MEME default is not as efficient as SLiMFinder at recovering known motifs (see Table 2). However, after the inclusion of both evolutionary weighting and masking out non-conserved residues, the performance of both methods are approximately equivalent. However, they don’t give identical results: SLiMFinder returns 3 motifs that MEME with weighting and masking fails to identify in the top ten motifs, namely SH3, 14-3-3_1 and RB; the latter motif was identified by the default MEME programme but lost in the modified version. However, MEME with weighting and masking does identify the NRBOX motif that would not have been discovered by either SLiMFinder or MEME default, indicating that the approach may be complementary to regular expression searching. SLiMFinder returns 41% of the true positives whilst MEME with evolutionary weighting and masking returns 72%. The false positive rate for SLiMFinder is 59% with 28% for MEME.

**Table 1:** **Performance using experimentally validated ELMs (dataset from [**[14]**]), searching for protein short linear motifs in disordered regions of proteins**

**ELM name**	**Number of proteins**	**Regular expression from ELM database**	**SLIMFinder with evolutionary weighting and RLC masking (Rank)**	**MEME default (Rank)**	**MEME with evolutionary weighting and RLC masking (Rank)**
TRG_ER_KDEL_1	12	[KRH][DENQ]EL	K.{0,2}DEL$ (1)	DEL (35)	DEL (1)
LIG_Dynein_DLC8_1	4	[KR].TQT	S.K.TQT (1)	K[AESV]TQ[TE][PD] (1)	[KV][SAE]TQT 91)
LIG_PCNA	13	Q.[ILM].[FHM][FHM]	[IL].S[FH]F (1)	Q.[SRT][IL][DM]SFF (1)	[LI].SFF (3)
MOD_SUMO	29	[VILAFP]K.[EDNGP]	[FIV]K.E (1)	[IV]K[QE]E[PE] (1)	[IV]KEE (1)
LIG_SH3_2	9	P.P.[KR]	P.P.R.{0,1}P (1)	-	-
LIG_CYCLIN_1	22	[RK].L.{0-1}[FYLIVMP]	RR.{0,1}L.{0,1}F (1)	[GE]L[St]R[ED]L.[KE][HLR]L (5)	K[KR][KR] (1)
LIG_CtBP	26	P.[DEN]L[VAST]	P[ILM]DL (1)	PLDLS (1)	PLDLS (1)
LIG_AP_DAE_1	8	[DE][DES].[F].[DE][LVIMFD]	D.F.F.S.P (1)	DDEF[GS][DE]FQ (1)	[GA]DF (1)
LIG_14-3-3_3	6	[RHK][STALV].[ST]. [PEDSIF]	S.P.S.T.P (3)	R[TS]NSA (65)	-
LIG_RB	25	[LI].C.[DE]	L.C.E (6)	LVCFE (1)	-
LIG_Clathr_ClatBox_1	15	l[ILM].[ILMF][DE]	L.{1,2}DL.{0,2}D (12)	[DE][ST][NSD]l[LI][DE][LF] (9)	[LG]L[DG]LD[SG](1)
LIG_14-3-3_1	4	R[FSWY].S.P	RS.S.P (3)	RS[IPRT]S[ALMT]P (29)	S[AI]S[ALE]P (1)
LIG_RGD	15	RGD	R.D.V (7)	RGD (6)	RGD (3)
LIG_HP1_1	6	P.V.[LM]	-	-	-
LIG_NRBOX	9	L.LL	-	[KN]H[AKP]LLS[RN]LL[RQ] (21)	L[KRS][QY]LL (1)
MOD_N-GLC_2	5	N.C	-	[FHIMY][NS][EANS][CE][VENS] [CEHRV][VAF][MKLV][EAGS][NE] (42)	-
TRG_lysEnd_APsAcLL	10	[DER]…L[LVI]	-	-	-

All proteins contain at least one experimentally determined motif instance. The regular expression that matches the annotated ELM regular expression is returned for each method along with its rank (in brackets). No result is indicated by a dash. Niall Haslam 24 October 2012 09:18.

**Table 2:** **Summary of performance using experimentally validated ELMs (see Table**1)

	**SLiMFinder**	**MEME default**	**MEME with weighting and RLC masking**
Number of First hits	8	6	9
Number in Top 10	12	9	11
Total	17	17	17
Percentage First Hit	47%	35%	53%
Percentage Top 10	71%	53%	65%

### Profile methods complement regular expression methods with a realistic biological discovery test dataset

Table 3 shows the results for the HPRD dataset, which represents a more realistic motif discovery example, since not all proteins contain a validated instance of the motif. Thus, this represents a typical question that would be asked by experimentalists trying to discover motifs in their protein-protein interaction data. In the case of HPRD SH2_GRB2 example, both weighting and masking were required to identify the motif, since analysis without either or without both failed to identify the motif (Additional file 1: Table S1). A summary of the findings of these HPRD search results (Table 4) clearly indicates the benefits of applying both weighting and masking. MEME with weighting and masking does not perform quite as well in terms of identifying the true motif in the top of the list (Table 4); but it is equivalent in terms of identifying the true motif within the top 10, identifying 4 motifs that SLiMFinder failed to identify in the top 10 (Table 3). SLiMFinder returns 21% of the true positives whilst MEME with evolutionary weighting and masking returns 9%. Again, this indicates that the two methods are complementary.

**Table 3:** Performance for MEME searching for short linear motifs in a realistic motif discovery scenario

**HPRDID**	**Hubgene symbol**	**Number of proteins (with motif)**	**Complex name**	**Motif name (from ELM)**	**Regular expression from ELM database**	**SLiMFinder with evolutionary weighting and RLC Masking**	**Meme default**	**MEME with evolutionary weighting RLC masking**
150	GRB2	164 (146)	Grb2	LIG_SH3	P.P	-	-	-
		164 (103)		LIG_SH2_GRB2	Y.N	Y.N[LMV] (3)	NK[NEK]KNRY[KV][DN]I (2)	KNRY[KPV][ND]ILP (1)
215	YWHAH	47 (13)	14-3-3 Eta	LIG_14-3-3_1	R[SFYW].S.p	[KR]S.S.P (1)	-	PKIHRSASEP (17)
350	CLTC	35 (15)	Clathrin, heavy polypeptide	LIG_Clathr_Clatbox_1	L[ILM].[ILMF][DE]	LLDL (4)	-	LLDL[EDM][DS][FA]QP (18)
453	CCNA2	25 (23)	Cyclin, A2	LIG_CYCLIN_1	[RK].L.{0,1}[FYLIVMP]	-	A[CK]R[RN]LFG (7)	SA[CK]R[NR]LFG (8)
607	FNTA	10 (2)	Farnessyltransferas e alpha subunit	MOD_ASX_betaOH_EGF	C[^DENQ][LIVM].$	-	C[DT]IS (30)	C[DT]IS (17)
627	IGTAS	15 (7)	Intergin alpha 5	LIG_RGD	RGD	-	KGDRGDA (25)	RG[DQ] (34)
1456	PCNA	65 (13)	Proliferating cell nuclear antigen	LIG_PCNA	Q.[ILM].[FHM][FHM]	Q.[IL].FF (1)	TL[YES]SFF (3)	TL[YES]SFF (2)
1574	RB1	110 (28)	Retinoblastoma 1	LIG_RB	[LI].C.[DE]	-	-	-
3288	PPARG	22 (15)	Peroxisome proliferator AR	LIG_NRBOX	L.LL	L.RLL (1)	HKILHRLLQ (4)	[LT][VS]HKLVQ[AL][IL] (1)
3334	DYNLL1	52 (5)	Dynien light chain 1	LIG_Dynien_DLC8_1	[KR].TQT	-	[MV]S[CY][DS]K[ES]TQTP (95)	KSTQT (10)
3786	NEDD4	28 (17)	NEDD4	LIG_WW_1	PP.Y	PP.Y (7)	PPAY (83)	PPPYSSI (2)
3833	TRAF6	22 (19)	TRAF6	LIG_TRAF6	.P.E.[FYWHDE].	-	-	-
4015	CTBP1	26 (14)	C-terminal binding protien	LIG_CtBP	P.[DEN]LVAST]	D.P[IL]D (6)	-	P[LI]DLS (1)
4946	CCNA1	20 (20)	Cyclin A1	LIG_CYCLIN_1	[RK].L.{0,1}[FYLIVMP]	-	A[CK]R[RN]LFG (3)	A[CK]R[RN]LFG (1)
5462	GIPC1	26 (7)	GIPC1	LIG_PDZ_1	.[ST].[VIL]$	S.V$ (1)	-	-
5639	YWHAG	206 (28)	14-3-3 gamma	LIG_14-3-3_1	R[SFYW].S.P	R.RS.S.S (1)	SRSRSRS[KR]SR (51)	[SK]SRSRS[RK]SR (30)
8968	EPS15	24 (10)	Eps15	LIG_EH	NPF	TNPF (1)	TNPF[LS](3)	TNPF (1)
9045	UBE2I	87 (78)	Ubiquitin conjugating enzyme E1	MOD_SUMO	[VILAFP]K.[EDNGP]	VK.E (2)	M[KM]VKDEY (18)	VEIVYE (7)
9347	GGA2	19 (2)	GGA2	LIG_AP_GAE_1	[DE][DES].F.[DE] [LVIMFD]	DDF.F.A (1)	D[DL]FG[GDE]F (6)	D[DL]FG[GDE]F (7)
9424	YAP1	15 (8)	YES associated protein	LIG_WW_1	PP.Y	-	[PL][PD]PPY (50)	H[CT][TY][LP]PPPY (6)

The HPRD dataset used, its name and the number of proteins in that dataset along with the ELM known to mediate some interactions with the protein hub are shown. The regular expression returned highest that matches the annotated ELM and its rank (in brackets) for SLiMFinder and MEME with and without RLC masking and evolutionary weighting are shown. No result is indicated with a dash. Niall Haslam 24 October 2012 09:19.

**Table 4:** **Summary of the results in a realistic motif discovery scenario (see Table**3**)**

	**SLiMFinder**	**MEME default**	**MEME with weighting and RLC masking**
Number returned	12	14	17
Number of first hits	7	0	5
Total	21	21	21
Number in Top Ten	12	6	12
Percentage Returned	76%	67%	81%
Percentage Top 10	57%	29%	57%
Percentage Top Hit	33%	0%	24%

Thus, the addition of the evolutionary weighting and RLC masking is able to increase the ability of MEME to identify the correct motif in the top 10 results returned. This indicates the likely benefits of including evolutionary weighting and RLC masking in *de novo* motif discovery, particularly for motifs lying in structurally disordered regions, which are strongly represented within both test datasets.

It might be expected that the motifs returned by MEME are richer, containing more information, since the profile representation has the potential to encode more information. Table 5 shows the SeqLogo representation of a sample of the motifs. Additional file 2: Table S2 indicates that for the ELM dataset, of motifs returned by both methods, for eight of them, MEME was more informative (motif less likely to occur by chance), one was equal, and for two SLiMFinder was more informative. For the more biologically realistic HPRD dataset (Additional file 3: Table S3), all 17 comparable motifs were more informative for MEME. This feature of MEME, that it tends in a realistic setting to return more informative motifs, may be valuable, since it may be detecting true subtleties that are missed by regular expression searching. However, we cannot exclude the possibility that it is simply over-fitting to the available data.

**Table 5:** **Sample of Sequence Logos from the MEME output from Table **1

**ELM name**	**MEME defined regular expression**	**SeqLogo from MEME**
Lig_Dynein	[KV][SAE]TQT	
Lig PCNA	[LI].SFF	
Lig Cyclin 1	K[KR][KR]	
Lig CtBP	PLDLS	
Lig KDEL	DEL	

In the example of Lig_Dynein the profile accurately captures the lack of flexibility in the final three positions. These three residues are all well conserved and contribute hydrogen bonds to the interaction. We examined the available structures using the ligplot software [31], where the profile allows Serine, Glutamic Acid and Alanine at position two in the motif. No structures were available for the case of Valine at position 1. In the case of Serine (PDB id: 1 F95) and Alanine (PDB id: 3E2B) at position two, there is no evidence of these residues contributing hydrogen bonds to the binding [32,33]. In the case of Glutamic Acid (PDB id: 2PG1) it does contribute a hydrogen bond to the interaction [34]. Thus, motifs with a charged rather than a small residue at position two may have a distinct mode of binding.

The approaches described here will be useful for proteomics experiments where the user expects that associations and interactions are mediated by short linear motifs. Accordingly, we have made scripts available to facilitate calculation of the weights for submission to MEME, as well as for the masking out of non-conserved residues at http://bioware.ucd.ie/meme.html. Developers interested in contributing to the further development of this freely available software are invited to apply for access to the subversion software repository. Applications of this approach should cite this paper, but also MEME [16]. Other tools employed such as IUPred [27], BLAST [35], Muscle [25], ClustalW [36] should be acknowledged as appropriate.

## Discussion

In the search for linear protein motifs the recent focus has centred on regular expressions. Whilst the methods that have transferred from DNA transcription factor searching such as MEME and NestedMica [37] have been applied to motif searching in proteins, profile-based methods have not typically been applied in SLiM databases such as ELM, phospho.ELM and MiniMotif. Regular expression based definitions have been preferred for a number of reasons, from ease of use to the fact that they can include subjective annotations from expert curators of the databases. However, in the move to more automated methods of protein linear motif discovery, there are a number of advantages to incorporating profile-based definitions, as these may increase the informativeness of the motifs under certain circumstances.

It is of interest to speculate on possible reasons why the MEME-based approach adopted here may give different results to regular expression approaches such as SLiMFinder. One possible reason is that certain motifs may evolve in a way that is best described by a regular expression, and therefore regular expressions will have more power to detect them, whilst other motifs evolve in a way that is more easily captured by a profile representation, with requirements for a specific subset of residues at certain positions that do not match the common ambiguity sets implemented in regular expression searching. We would have anticipated that profile representations will become more powerful for motifs with many occurrences, since the profile definitions are more approximate when there are only a few sequences, but we find no clear suggestions to date that this is the case (Tables 1 and 2). The two search strategies differ in another subtle way: with the MEME approach, the sequence weighting occurs before motif discovery, whereas with SLiMFinder motifs are selected within the entire protein dataset and then ranked afterwards, on the basis of statistical support. It is possible that this may favour SLiMFinder whenever a motif is found in only a small subset of the related proteins. In the 8 test datasets where the motif is found in less than 30% of the interacting proteins, SLiMFinder finds the correct motif more often than MEME, 5 times compared to 3 times.

The discovery of linear motifs in interaction sets and proteomics experiments is only one step in the process of determining the functionality of proteins or sets of proteins in an interaction network. Profile-based methods will be useful in the process of searching for further instances of motifs identified in one experiment. Given the success of profile-based searching methods in complementing sequence based searching in domain recognition, [38,39], we anticipate that profile-based approaches should also take their place alongside regular expression methods in SLiM identification (e.g. SLiMSearch [40]).

## Abbreviations

SLiM: Short linear motif; ELM: Eukaryotic linear motif; HPRD: Human protein reference database; RLC: Relative local conservation; UPC: Unrelated protein clusters.

## Competing interests

The authors declare that they have no competing interests.

## Author’s contributions

Conception, study design and writing: NH and DS. Coding and implementation: NH. Both authors read and approved the final manuscript.

## Supplementary Material

Additional file 1 **Table S1.** Summary of Performance using a realistic motif discovery scenario, searching for protein short linear motifs in disordered regions of proteins with MEME. Click here for file

Additional file 2 **Table S2.** Probability that a motif occurs by chance as an indicator of information content of the motif representation. Pmotif scores [15] relate to results from Table 1. Click here for file

Additional file 3 **Table S3.** Probability that a motif occurs by chance as an indicator of information content of the motif representation. Pmotif scores [15] relate to results from Table 2. Click here for file

## References

[B1] Neduva V Russell RB Linear motifs: evolutionary interaction switches FEBS Lett 2005 579 3342 3345 10.1016/j.febslet.2005.04.005 15943979

[B2] Letunic I Doerks T Bork P SMART 6: recent updates and new developments Nucleic Acids Res 2009 37 D229 D232 10.1093/nar/gkn808 18978020 PMC2686533

[B3] Dinkel H Michael S Weatheritt RJ Davey NE Van Roey K Altenberg B Toedt G Uyar B Seiler M Budd A ELM–the database of eukaryotic linear motifs Nucleic Acids Res 2012 40 D242 251 10.1093/nar/gkr1064 22110040 PMC3245074

[B4] Miller MLL Jensen LJJ Diella F Jørgensen C Tinti M Li L Hsiung M Parker SA Bordeaux J Sicheritz-Ponten T Linear motif atlas for phosphorylation-dependent signaling Sci Signal 2008 1 ra2 10.1126/scisignal.1159433 18765831 PMC6215708

[B5] Gibson TJ Cell regulation: determined to signal discrete cooperation Trends Biochem Sci 2009 34 471 482 10.1016/j.tibs.2009.06.007 19744855

[B6] Rajasekaran S Balla S Gradie P Gryk MRR Kadaveru K Kundeti V Maciejewski MWW Mi T Rubino N Vyas J Schiller MRR Minimotif miner 2nd release: a database and web system for motif search Nucleic Acids Res 2008 10.1093/nar/gkn865 PMC2686579 18978024

[B7] Dinkel H Chica C Via A Gould CM Jensen LJ Gibson TJ Diella F Phospho.ELM: a database of phosphorylation sites--update 2011 Nucleic Acids Res 2010 39 D261 D267 21062810 10.1093/nar/gkq1104 PMC3013696

[B8] Gnad F Ren S Cox J Olsen JV Macek B Oroshi M Mann M PHOSIDA (phosphorylation site database): management, structural and evolutionary investigation, and prediction of phosphosites Genome Biol 2007 8 11 R250 10.1186/gb-2007-8-11-r250 18039369 PMC2258193

[B9] Ward JJ Sodhi JS McGuffin LJ Buxton BF Jones DT Prediction and functional analysis of native disorder in proteins from the three kingdoms of life J Mol Biol 2004 337 635 645 10.1016/j.jmb.2004.02.002 15019783

[B10] Gould CM Diella F Via A Puntervoll P Gemünd C Chabanis-Davidson S Michael S Sayadi A Bryne JC Chica C ELM: the status of the 2010 eukaryotic linear motif resource Nucleic Acids Res 2010 38 D167 180 10.1093/nar/gkp1016 19920119 PMC2808914

[B11] Via A Gould C Gemund C Gibson T Citterich MH A structure filter for the Eukaryotic Linear Motif Resource BMC Bioinforma 2009 10 10.1186/1471-2105-10-351 PMC2774702 19852836

[B12] Down TA Bergman CM Su J Hubbard TJ Large-scale discovery of promoter motifs in Drosophila melanogaster PLoS Comput Biol 2007 3 e7 10.1371/journal.pcbi.0030007 17238282 PMC1779301

[B13] Bailey TL Discovering novel sequence motifs with MEME Current protocols in bioinformatics/editoral board, Andreas D Baxevanis [et al] 2002 **Chapter 2:**Unit 2.4 10.1002/0471250953.bi0204s00 18792935

[B14] Neduva V Russell RB DILIMOT: discovery of linear motifs in proteins Nucleic Acids Res 2006 34 10.1093/nar/gkl159 PMC1538856 16845024

[B15] Davey NE Haslam NJ Shields DC Edwards RJ SLiMFinder: a web server to find novel, significantly over-represented, short protein motifs Nucleic Acids Res 2010 1 6 10.1093/nar/gkq440 PMC2896084 20497999

[B16] Bailey TL Boden M Buske FA Frith M Grant CE Clementi L Ren J Li WW Noble WS MEME SUITE: tools for motif discovery and searching Nucleic Acids Res 2009 37 W202 208 10.1093/nar/gkp335 19458158 PMC2703892

[B17] Diella F Haslam N Chica C Budd A Michael S Brown NP Trave G Gibson TJ Understanding eukaryotic linear motifs and their role in cell signaling and regulation Front Biosci 2008 13 6580 6603 18508681 10.2741/3175

[B18] Crooks GE Hon G Chandonia JM Brenner SE WebLogo: a sequence logo generator Genome Res 2004 14 1188 1190 10.1101/gr.849004 15173120 PMC419797

[B19] Davey NE Shields DC Edwards RJ Masking residues using context-specific evolutionary conservation significantly improves short linear motif discovery Bioinformatics (Oxford, England) 2009 25 443 450 10.1093/bioinformatics/btn664 19136552

[B20] Consortium TU The Universal Protein Resource (UniProt) in 2010 Nucleic Acids Res 2010 38 D142 D148 19843607 10.1093/nar/gkp846 PMC2808944

[B21] Fuxreiter M Tompa P Simon I Local structural disorder imparts plasticity on linear motifs Bioinformatics (Oxford, England) 2007 23 950 956 10.1093/bioinformatics/btm035 17387114

[B22] Davey NE Van Roey K Weatheritt RJ Toedt G Uyar B Altenberg B Budd A Diella F Dinkel H Gibson TJ Attributes of short linear motifs Mol Biosyst 2011 8 268 281 21909575 10.1039/c1mb05231d

[B23] Hubbard TJP Aken BL Ayling S Ballester B Beal K Bragin E Brent S Chen Y Clapham P Clarke L Ensembl 2009 Nucleic Acids Res 2009 37 D690 D697 10.1093/nar/gkn828 19033362 PMC2686571

[B24] Edwards RJ Davey NE Shields DC SLiMFinder: A Probabilistic Method for Identifying Over-Represented, Convergently Evolved, Short Linear Motifs in Proteins PLoS One 2007 2 10 e967 10.1371/journal.pone.0000967 17912346 PMC1989135

[B25] Edgar RC MUSCLE: a multiple sequence alignment method with reduced time and space complexity BMC Bioinforma 2004 5 113 10.1186/1471-2105-5-113 PMC517706 15318951

[B26] Davey NE Edwards RJ Shields DC The SLiMDisc server: short, linear motif discovery in proteins Nucleic Acids Res 2007 35 W455 459 10.1093/nar/gkm400 17576682 PMC1933137

[B27] Dosztányi Z Csizmok V Tompa P Simon I IUPred: web server for the prediction of intrinsically unstructured regions of proteins based on estimated energy content Bioinformatics (Oxford, England) 2005 21 3433 3434 10.1093/bioinformatics/bti541 15955779

[B28] Neduva V Russell R Linear motifs: evolutionary interaction switches FEBS Lett 2005 579 3342 3345 10.1016/j.febslet.2005.04.005 15943979

[B29] Gould CM Diella F Via A Puntervoll P Gemund C Chabanis-Davidson S Michael S Sayadi A Bryne JC Chica C Others: ELM: the status of the 2010 eukaryotic linear motif resource Nucleic Acids Res 2010 38 D167 10.1093/nar/gkp1016 19920119 PMC2808914

[B30] Prasad TS Kandasamy K Pandey A Human Protein Reference Database and Human Proteinpedia as discovery tools for systems biology Methods Mol Biol 2009 577 67 79 10.1007/978-1-60761-232-2_6 19718509

[B31] Laskowski RA Swindells MB LigPlot+: multiple ligand-protein interaction diagrams for drug discovery J Chem Inf Model 2011 51 2778 2786 10.1021/ci200227u 21919503

[B32] Fan J Zhang Q Tochio H Li M Zhang M Structural basis of diverse sequence-dependent target recognition by the 8 kDa dynein light chain J Mol Biol 2001 306 97 108 10.1006/jmbi.2000.4374 11178896

[B33] Benison G Karplus PA Barbar E The interplay of ligand binding and quaternary structure in the diverse interactions of dynein light chain LC8 J Mol Biol 2008 384 954 966 10.1016/j.jmb.2008.09.083 18948118 PMC4294432

[B34] Williams JC Roulhac PL Roy AG Vallee RB Fitzgerald MC Hendrickson WA Structural and thermodynamic characterization of a cytoplasmic dynein light chain-intermediate chain complex Proc Natl Acad Sci U S A 2007 104 10028 10033 10.1073/pnas.0703614104 17551010 PMC1885999

[B35] Altschul S Gish W Miller W Myers E Lipman D Basic local alignment search tool J Mol Biol 1990 215 403 410 2231712 10.1016/S0022-2836(05)80360-2

[B36] Sievers F Wilm A Dineen D Gibson TJ Karplus K Li W Lopez R McWilliam H Remmert M Soding J Fast, scalable generation of high-quality protein multiple sequence alignments using Clustal Omega Mol Syst Biol 7 539 21988835 10.1038/msb.2011.75 PMC3261699

[B37] Doğruel M Down Ta Hubbard TJ NestedMICA as an ab initio protein motif discovery tool BMC Bioinforma 2008 9 19 10.1186/1471-2105-9-19 PMC2267705 18194537

[B38] Finn RD Clements J Eddy SR HMMER web server: interactive sequence similarity searching Nucleic Acids Res 2011 39 W29 37 10.1093/nar/gkr367 21593126 PMC3125773

[B39] Finn RD Tate J Mistry J Coggill PC Sammut SJ Hotz H-R Ceric G Forslund K Eddy SR Sonnhammer ELL Bateman A The Pfam protein families database Nucleic Acids Res 2008 36 D281 D288 10.1093/nar/gkn226 18039703 PMC2238907

[B40] Davey NE Haslam NJ Shields DC Edwards RJ SLiMSearch 2.0 : biological context for short linear motifs in proteins Nucleic Acids Res 2011 Webserver 1–5 10.1093/nar/gkr402 PMC3125787 21622654

