# Supplementary material for: Profile-based short linear protein motif discovery
Source: BMC Bioinformatics. 2012 May 18;13:104. doi: 10.1186/1471-2105-13-104 (PMC3534220; doi:10.1186/1471-2105-13-104)
Supplement: Additional file 1 — Table S1. Summary of Performance using a realistic motif discovery scenario, searching for protein short linear motifs in disordered regions of proteins with MEME. [file 1471-2105-13-104-S1.doc]

Supplementary Table 1: Summary of Performance using a realistic motif discovery scenario, searching for protein short linear motifs in disordered regions of proteins with MEME.
