# Supplementary material for: Profile-based short linear protein motif discovery
Source: BMC Bioinformatics. 2012 May 18;13:104. doi: 10.1186/1471-2105-13-104 (PMC3534220; doi:10.1186/1471-2105-13-104)
Supplement: Additional file 3 — Table S3. Probability that a motif occurs by chance as an indicator of information content of the motif representation. Pmotif scores [15] relate to results from Table 2. [file 1471-2105-13-104-S3.doc]

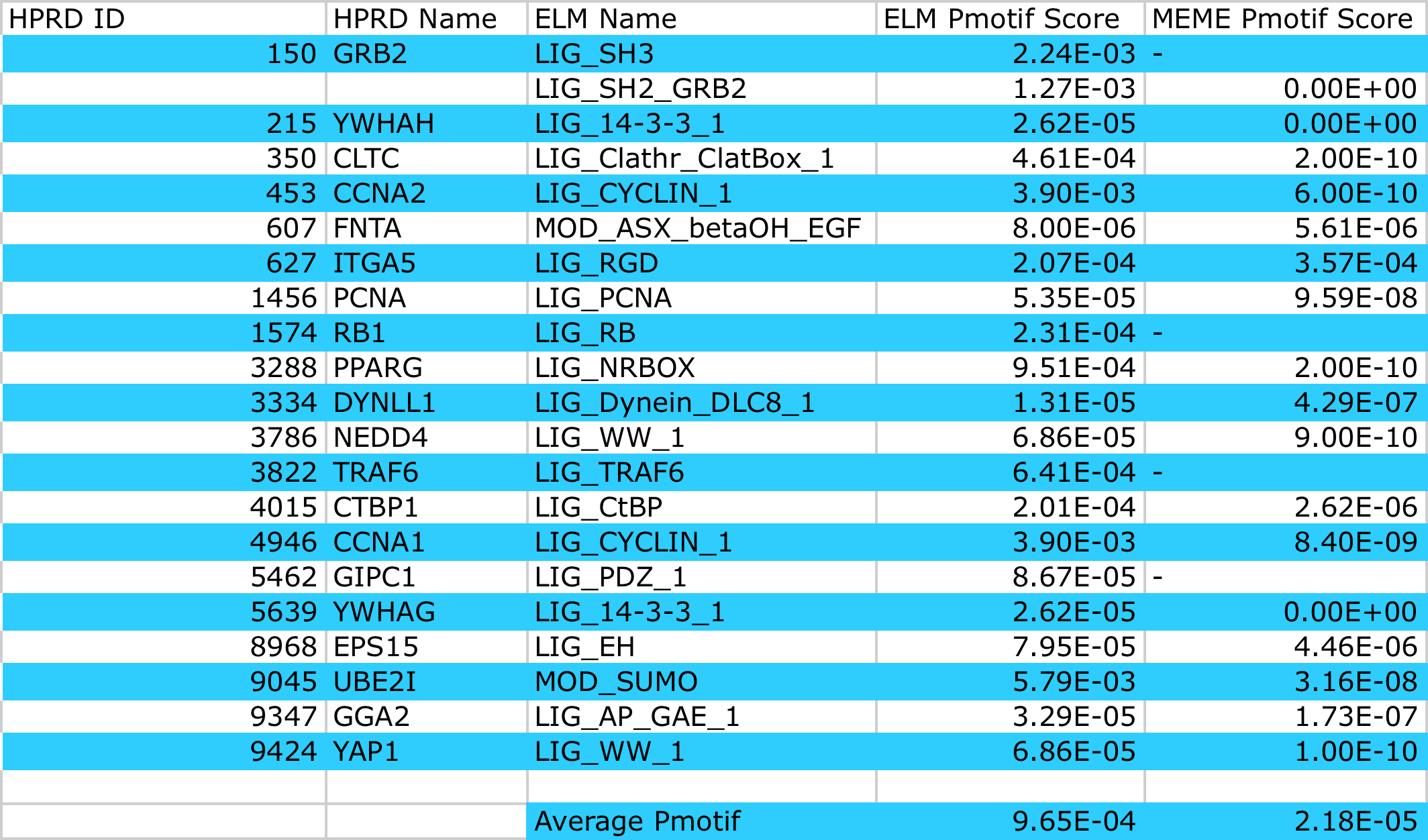


Supplementary Table 3: Probability that a motif occurs by chance as an indicator of information content of the motif representation. Pmotif scores [15] relate to results from Table 2.
